# Frontline Nurse Managers' Views of Person‐Centred Management in a Private Hospital: A Qualitative Study

**DOI:** 10.1002/nop2.70753

**Published:** 2026-08-13

**Authors:** Furkan Cihat Arici, Arzu Kader Harmanci Seren

**Affiliations:** ^1^ Institute of Graduate Education Istanbul Okan University Istanbul Türkiye; ^2^ Koç University Hospital Istanbul Türkiye; ^3^ Department of Nursing, Faculty of Health Sciences Fenerbahce University Istanbul Türkiye; ^4^ Atatürk Mah. Ataşehir Bulvarı, Metropol İstanbul İstanbul Türkiye

## Abstract

**Aim:**

To explore the perceptions and experiences of frontline nurse managers regarding the person‐centred management approach.

**Design:**

A qualitative study.

**Methods:**

The study adopted a qualitative approach to gain an in‐depth understanding of frontline nurse managers' experiences. Data were collected through semi‐structured, in‐depth interviews between February and June 2024, and content analysis techniques were used to extract the essence of the data during analysis.

**Results:**

Analysis of the interviews revealed three main domains reflecting the frontline nurse managers' perceptions and experiences. First, participants described employing diverse leadership styles to facilitate person‐centred management. Second, they detailed specific person‐centred management practices that they perceived as improving both employees and patient outcomes. Finally, managers highlighted the challenges they faced in implementing person‐centred management and shared the adaptive coping strategies they used to maintain person‐centred care.

**Conclusion:**

To achieve a person‐centred management approach, frontline nurse managers apply various leadership styles to support patient, employee, and organizational outcomes. Furthermore, their ability to navigate institutional challenges through effective coping strategies is essential for sustaining person‐centred environments.

**Impact:**

This study provides actionable insights into how frontline nurse managers experience and navigate person‐centred management. The findings suggest that adopting leadership styles and effective coping strategies can be instrumental in navigating institutional challenges. By understanding these dynamics, healthcare organizations can cultivate a supportive management culture that facilitates and sustains the delivery of high‐quality, person‐centred care.

**Reporting Method:**

This study is reported in accordance with the COREQ guideline.

**Patient or Public Contribution:**

This study did not include patient or public involvement in its design, conduct, or reporting.

## 1 Introduction

In the contemporary healthcare environment, marked by constant change and growing complexity, nurse well‐being has shifted from an individual goal to a strategic need for sustainable healthcare systems (Bond et al. 2025). Global crises and the inherent stress of clinical settings have shown that leadership based solely on administrative control is insufficient to ensure the continuity of nursing care (Leclerc et al. 2021). As a result, a shift is underway from technical‐managerial controls to Person‐centred Management (PCM) approaches, which now form the foundation of sustainable organizational success (Lepeley 2020, 2023).

Person‐centred care (PCC), widely regarded as the standard for healthcare quality (Abugre and Bhengu 2024), hinges on fostering a conducive work environment for nurses providing direct care. Despite current literature demonstrating that organizational support and co‐management enhance nurse well‐being (Choi et al. 2021; Nielsen et al. 2025), the practical implementation of this theoretical framework is hindered by multidisciplinary dynamics. During the transition to a person‐centred institutional culture, variable physician attitudes towards this model (Liang et al. 2023) and inadequate staffing to support nursing teamwork in clinical settings (Baek et al. 2023) complicate multidisciplinary alignment. In this clinical setting, characterized by interprofessional conflicts, the mechanisms by which supportive managerial practices overcome this resistance and foster a healthy climate remain underexplored in the literature (Anker‐Hansen et al. 2025; Backman, Lövheim, et al. 2021).

Specifically, nurse managers translate organizational strategies into clinical safety. While current literature emphasizes leadership's role in reducing moral distress and turnover (Howle et al. 2025), little research explains how these essential soft skills, which shape organizational culture, are developed through daily management. This leaves a critical research gap regarding how frontline nurse managers operationalize PCM within high‐pressure clinical realities.

The significance of this study for nursing practice lies in its potential to explicitly address this gap. By exploring the lived experiences of frontline leaders, this research translates theoretical leadership concepts into actionable, evidence‐based strategies. Uncovering the practical challenges and facilitators of PCM provides healthcare administrators with insights to redesign support systems, mitigate nurse burnout, and improve workforce retention. Ultimately, this study aims to demonstrate that fostering a genuine PCM culture is not just an administrative goal, but a strategy to sustain high‐quality nursing care.

## 2 Background

The operationalization of PCM requires a conceptual framework consistent with the core principles of person‐centred practice (McCormack and McCance 2016; McCance and McCormack 2025). Unlike other positive management models, such as transformational or authentic leadership, PCM focuses not merely on guiding staff but on the mutual recognition of personhood and the cultivation of relationships across all organizational levels (Cardiff et al. 2018). By implementing this model, nurse managers build a workplace culture grounded in respect; this supportive environment directly facilitates the staff's ability to deliver PCC to patients (Lynch et al. 2018). In this context, PCC acts as a systemic framework that shapes clinical practice and structures daily operations.

Although PCC and PCM share an underlying humanistic philosophy, they operate at distinct conceptual and operational levels. PCC focuses primarily on the clinical delivery of care, prioritizing the individual needs, preferences, and personhood of patients (McCormack and McCance 2016; McCance and McCormack 2025). In contrast, PCM functions at the leadership and organizational levels, encompassing management practices that recognize and nurture the personhood, well‐being, and professional growth of employees (Lepeley 2020, 2023). PCM serves as the foundational infrastructure for PCC; by cultivating a supportive, psychologically safe, and empowering work environment, PCM enables, sustains, and operationalizes person‐centred care practices within nursing services. This systemic synergy is conceptualized through the ‘manager‐employee‐patient triad’, a chain of influence in which the nurse manager's person‐centred leadership creates a supportive environment for the nursing staff. This support directly enhances the staff's capacity and motivation to deliver high‐quality, person‐centred care to the patient, representing the systemic link where managerial support acts as a prerequisite for improved patient outcomes.

Outcomes of PCM include compassionate communication, talent development, and employee well‐being (Lepeley 2023). This framework, increasingly prevalent in broader organizational and societal contexts, is also gaining momentum in healthcare settings (Sherman et al. 2024). In PCM, individual differences are viewed not as organizational obstacles, but as prerequisites for creating a supportive work environment (Lepeley 2020). Creating this environment requires a person‐centred climate, a shared vision, effective resource management, and structural empowerment (Wang and Dewing 2021). Institutionalizing this framework necessitates a cultural shift from rigid hierarchical structures to environments characterized by compassion, talent management, and psychological safety (Abdelaliem et al. 2025; Lambert et al. 2024). However, without shared understanding and supportive organizational structures, the implementation of PCM remains complex. It can unintentionally lead to staff burnout and counteract the core objectives of PCM practice.

The creation of a person‐centred climate is, as argued, contingent upon facilitative and positive leadership behaviours (Backman, Lövheim, et al. 2021; Backman, Sjögren, et al. 2021). When managers actively demonstrate person‐centred leadership, it is associated with enhanced psychological safety in clinical settings (Cho and Steege 2025). Moreover, these dynamics play a pivotal role in shaping patient safety. Research indicates that work environments characterized by high levels of psychological safety foster nurse retention and mitigate incidents of missed care, thereby actively supporting overall clinical outcomes (Backman, Lövheim, et al. 2021; Backman, Sjögren, et al. 2021; Cho and Steege 2025). Ultimately, this person‐centred approach empowers clinical staff to take initiative and serve as role models within their units (She et al. 2025).

Despite extensive research on the multifaceted benefits of PCM, the adoption of such practices in clinical settings depends on the harmonization of structural barriers and facilitators. Although the benefits of PCM are well‐documented, its clinical adoption depends on navigating structural barriers. Research suggests that excessive workloads, hierarchical pressures, and negative organizational climates often impede managers' ability to exhibit person‐centred leadership (Abugre and Bhengu 2024; Howle et al. 2025). Conversely, shared governance mechanisms that involve staff in decision‐making and compassion‐based organizational interventions that grant managers autonomy emerge as strategic facilitators that enable the overcoming of these barriers (Nielsen et al. 2025).

A thorough review of current literature consistently highlights the importance of person‐centred approaches for achieving a sustainable nursing workforce and high‐quality care outcomes. A substantial body of research has extensively explored the positive outcomes of organizational support and compassion‐based interventions in promoting nurses' well‐being within a conducive work environment (Bond et al. 2025; Nielsen et al. 2025). Moreover, supportive leadership is crucial in helping nurses navigate feelings of worthlessness, particularly in high‐pressure clinical environments (Howle et al. 2025).

Even with ample evidence supporting PCM, most research focuses on the perspectives of senior nurse managers or direct‐care clinical nurses (Abugre and Bhengu 2024). This leaves a gap in research on frontline nurse managers, who ensure continuity of care and translate large‐scale decisions into daily practice.

To address that gap, this qualitative study directly examines PCM from the perspectives of frontline nurse managers, aiming to illuminate the divide between management vision and clinical reality. By uncovering the practical challenges and facilitators of PCM, the findings provide actionable insights for healthcare administrators to redesign support systems, such as equitable reward mechanisms and leadership training. Ultimately, it offers actionable insights tailored for nursing administrators to support frontline leaders and maintain a sustainable, healthy work environment.

## 3 Methods

### 3.1 Aim

This study aimed to explore frontline nurse managers' experiences of the person‐centred management approach in a private hospital in Türkiye.

### 3.2 Design

In this study, a qualitative descriptive research design was adopted to examine, in depth and from their own perspectives, the person‐centred management experiences of frontline nurse managers in nursing services. According to Polit and Beck (2018), qualitative descriptive studies are the optimal approach in this case. These studies offer a comprehensive overview by delineating the participants' experiences in their original form. The authors demonstrate a commitment to maintaining the participants' language and eschewing theoretical abstractions. Concurrently, they do not adhere rigidly to any philosophical tradition. Reporting of the collected qualitative content followed the COREQ guidelines (Tong et al. 2007) and the completed COREQ checklist is provided as Supplementary File 1.

### 3.3 Participants

The study was conducted between February and June 2024, in a Planetree‐certified and Joint Commission‐accredited private hospital in Türkiye. It has a high‐volume, dynamic clinical environment (e.g., intensive care units, operating rooms) that provides an optimal context for evaluating PCM. To ensure in‐depth data collection and contextual validity through thick description, convenience sampling was employed. The inclusion criteria required participants to hold at least a bachelor's degree in nursing and to have at least 1 year of active experience in a frontline managerial position. Following the acquisition of ethics committee and institutional approvals, the recruitment process began. At the time of the study, there were 36 frontline nurse managers employed at the institution. Eleven managers were excluded before recruitment (three were unavailable due to long‐term leave, and eight did not meet the educational inclusion criteria), leaving an eligible pool of 25 managers. Consistent with the convenience sampling approach, the first author consecutively approached potential participants from this pool based on their daily availability, shift schedules, and operational workload. In total, 22 managers were approached; only one declined participation, expressing apprehension and discomfort regarding the audio recording process. Ultimately, data saturation was achieved with a final sample of 21 managers. At this point, recruitment was concluded, and the remaining three eligible managers were not approached. No participants dropped out after the interviews commenced.

### 3.4 Researcher Characteristics and Reflexivity

In qualitative research, the researcher serves as the primary instrument for data collection and analysis. In this study, data collection was conducted exclusively by the primary researcher (first author), while both researchers (first and second authors) analysed the data collaboratively. Furthermore, both authors held multiple roles in patient care and management, as well as administrative and academic roles. The triangulation of researcher roles, encompassing clinician, administrator, and academic functions, facilitated a comprehensive understanding of the phenomenon's practical and theoretical facets. This multifaceted approach contributed to the study's analytical rigour.

However, conducting insider research inherently introduces risks of managerial power asymmetry and social‐desirability bias. To explicitly control for this and mitigate potential bias in data collection, methodological boundaries were established. No participants were in a direct subordinate reporting line to the researcher conducting the interviews. Before data collection, written informed consent was obtained, with explicit guarantees of strict confidentiality. Furthermore, at the beginning of each interview and throughout, researchers frequently reiterated their role as independent researchers, assuring participants that their candid responses would remain completely anonymous and would have absolutely no bearing on their professional evaluations.

Alongside these procedural safeguards, reflexivity strategies were actively employed. Regular reflective debriefing sessions were conducted to critically examine how the researchers' own backgrounds, values, and professional roles might influence the research process. This comprehensive methodological approach enabled researchers to manage their insider status and cultivate a secure environment, allowing them to capture participants' authentic and sometimes dissonant experiences rather than mere compliant accounts (Polit and Beck 2018).

### 3.5 Data Collection

The present study utilized a socio‐demographic questionnaire comprising six items developed by the researchers, alongside a semi‐structured interview guide consisting of 10 questions (Table 1). To ensure the validity of the interview guide, it was reviewed by a multidisciplinary expert panel: two faculty members specializing in nursing management, a physician actively involved in person‐centred care accreditation, and a specialist nurse serving as a person‐centred care coordinator. Based on their feedback, no substantial revisions were required. Subsequently, a pilot study was conducted with two frontline nurse managers who met the inclusion criteria. No issues regarding the clarity of the questions were identified, and no modifications were made. The data obtained from these pilot interviews were excluded from the main study analysis.

**TABLE 1:** Semi‐structured data collection form.

Introduction	What does being ‘person‐centred’ mean to you?
	What does a person‐centred approach evoke in a hospital setting?
Transition	What does the concept of person‐centred management mean to you?
Key questions	What does person‐centred nursing services management mean to you?
	What do you think are the characteristics of a person‐centred nurse manager?
	What kinds of practices are implemented in your institution regarding person‐centred management?
	What can institutions do to ensure person‐centred management?
	What can nurse managers do for person‐centred management?
	What are the benefits of person‐centred management?
	What is needed for the easy implementation of person‐centred management?
	What are the obstacles to person‐centred management?
Closing	If you have any experience with person‐centred management, can you share it?
	Do you have any additional opinions you would like to add regarding person‐centred management?

Data were collected through individual face‐to‐face, in‐depth interviews between February and June 2024. Prior to the sessions, participants were fully informed about the research topic, the nature of qualitative research, and that the interviews would be audio‐recorded, after which they provided both verbal and written informed consent. With the participants' explicit permission, all interviews were audio‐recorded using a digital voice recorder to ensure complete and accurate capture of the dialogue. Following each session, the audio recordings were transcribed verbatim. All interviews were conducted by the first author at times most convenient for the managers' clinical schedules. To ensure maximum privacy and prevent external interruptions, the sessions were held in a quiet, isolated hospital room with restricted access. In unavoidable instances where a participant's hospital Digital Enhanced Cordless Telecommunications (DECT) phone rang due to clinical urgencies, the recording was temporarily paused, and these interruptions were rigorously documented in the interviewer's diary.

During the interviews, the researcher utilized probing questions (e.g., ‘Could you elaborate on that?’, ‘Can you provide a specific example from your unit?’) to deepen the dialogue and elicit richer data. The interview durations ranged from 32 to 63 min. Data collection continued concurrently with preliminary analysis until data saturation was reached. To confirm saturation, three supplementary interviews were conducted. With no new codes emerging, these final interviews concluded the data collection process.

### 3.6 Data Analysis

All interviews were conducted in Turkish to allow participants to express their experiences naturally. Following verbatim transcription in Turkish by the first researcher and subsequent verification by the second researcher, the data were managed and analysed using MAXQDA 22.0 software. A qualitative content analysis approach was employed to evaluate the interview records systematically. To preserve cultural nuances and contextual meaning, the entire data analysis process was also conducted in Turkish. Both researchers independently immersed themselves in the data, extracted meaningful units, and assigned initial codes. Any coding discrepancies were systematically resolved through joint discussions and consensus meetings (Polit and Beck 2018). To maintain methodological consistency, the agreed‐upon codes were compared for similarities and differences and grouped into sub‐domains and domains. Finally, the resulting domains were cross‐checked with the participants' original narratives to ensure descriptive accuracy. Once the analysis was complete, the selected representative quotations were translated into English with the assistance of a professional bilingual translator. To ensure conceptual equivalence, these translations were subsequently reviewed and cross‐verified by the second researcher, who is fully bilingual in Turkish and English.

### 3.7 Ethical Considerations

The study was conducted in strict accordance with the principles of the Declaration of Helsinki. Ethical approval was granted by the Ethics Committee of Koç University (Approval No: 2024.030.IRB3.013, Date: January 26, 2024). In addition to ethical clearance, formal institutional permission was obtained from the participating hospital before data collection. All participants were provided with detailed information regarding the study's aim, procedures, and their rights. Both verbal and written informed consent was obtained from all frontline nurse managers before the interviews. Participants were assured of their absolute anonymity, the strict confidentiality of their data, and their right to withdraw from the study at any time without any consequences.

### 3.8 Rigour and Trustworthiness

Lincoln and Guba's proposed steps were followed to establish rigour and reliability (Lincoln and Guba 1986). To ensure credibility and consistency, a single experienced researcher conducted all flexibly scheduled interviews using an expert‐validated and pilot‐tested semi‐structured guide (Table 1). Reflexive journaling and consistent question sequencing were maintained throughout. Interviews continued until data saturation was reached, as confirmed by three subsequent interviews. Dependability and confirmability were strengthened by maintaining a detailed audit trail of methodological decisions and conducting member checking with a subset of participants (*n*: 7) via email to verify the resonance of the preliminary findings. For analytical rigour, two researchers engaged in independent parallel coding of the transcripts. Any discrepancies in code generation or categorization were resolved through iterative consensus meetings. Finally, the study adhered to the COREQ checklist to guarantee transparent reporting (Tong et al. 2007).

## 4 Findings

### 4.1 Characteristics of Participants

This qualitative study was conducted with 21 frontline nurse managers (Table 2) working at a private foundation hospital with a person‐centred care certificate. To safeguard the confidentiality of the study's participants, the interviews were systematically coded as P1–P21 during data analysis. The study's participants were exclusively female, with an average age of 39.23 years (min: 31, max: 48). A greater proportion of the participants, specifically 57.14%, were married. Regarding educational attainment, most participants (71.42%) held a bachelor's degree. The mean duration of nursing experience was 17.23 years (min: 10, max: 28). Regarding current management positions, 61.90% of participants held charge nurse roles. The size of the managed units varied, with an average total number of employees per unit of 32.10 (min: 5, max: 100) and an average number of nurses of 20.67 (min: 3, max: 70). The in‐depth interviews conducted during the data collection process had an average duration of 43.28 min (min: 32, max: 63).

**TABLE 2:** Participants' characteristics.

Participant code	Age	Sex	Marital status	Graduation degree	Years of experience in the nursing profession	Current management position	Years of experience as a manager	Number of employees in the unit	Number of nurses in the team	Interview duration
P1	40	Woman	Single	Bachelor's Degree	18	Clinic Supervisor	10	19	5	52 min
P2	40	Woman	Married	Bachelor's Degree	18	Charge Nurse	14	19	11	44 min
P3	43	Woman	Married	Bachelor's Degree	18	Charge Nurse	10	62	24	49 min
P4	38	Woman	Married	Master's Degree Graduation	17	Clinic Supervisor	4	16	16	36 min
P5	37	Woman	Single	Bachelor's Degree	14	Clinic Supervisor	8	50	30	51 min
P6	44	Woman	Single	Bachelor's Degree	25	Clinic Supervisor	13	100	70	37 min
P7	44	Woman	Married	Bachelor's Degree	22	Clinic Supervisor	14	61	46	34 min
P8	49	Woman	Married	Bachelor's Degree	27	Clinic Supervisor	23	46	30	45 min
P9	33	Woman	Single	Master's Degree Graduation	11	Charge Nurse	6	12	10	39 min
P10	43	Woman	Single	Bachelor's Degree	19	Charge Nurse	10	24	16	63 min
P11	44	Woman	Married	Bachelor's Degree	20	Charge Nurse	10	16	12	57 min
P12	39	Woman	Married	Bachelor's Degree	17	Charge Nurse	8	22	16	33 min
P13	48	Woman	Single	Bachelor's Degree	28	Clinic Supervisor	10	32	24	53 min
P14	33	Woman	Married	Master's Degree Graduation	10	Charge Nurse	3	15	4	32 min
P15	39	Woman	Married	Bachelor's Degree	16	Charge Nurse	11	25	18	37 min
P16	35	Woman	Married	Bachelor's Degree	14	Charge Nurse	2	5	3	39 min
P17	32	Woman	Single	Master's Degree Graduation	10	Clinic Supervisor	5	47	32	45 min
P18	35	Woman	Single	Master's Degree Graduation	13	Charge Nurse	6	29	17	41 min
P19	38	Woman	Married	Master's Degree Graduation	18	Charge Nurse	12	21	17	44 min
P20	31	Woman	Married	Bachelor's Degree	10	Charge Nurse	2	20	15	35 min
P21	39	Woman	Single	Bachelor's Degree	17	Charge Nurse	9	33	18	43 min

### 4.2 Domains

**FIGURE 1:**
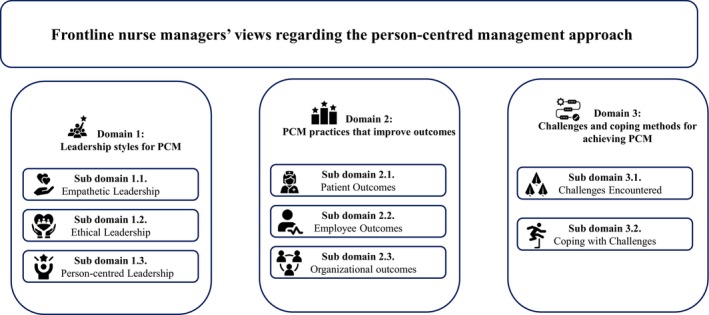
Domains and sub domains.

As a result of content analysis of data from individual in‐depth interviews with frontline nurse managers, 3 main domains and 8 sub‐domains were identified (Table 3, Figure 1). Representative quotations supporting the identified domains and sub‐domains are present within the text, while additional illustrative quotations are provided in Supplementary File 2.

**TABLE 3:** Domains and subdomains.

Domain	Sub domain
1. Leadership styles for person‐centred management	1.1. Empathetic leadership
	1.2. Ethical leadership
	1.3. Person‐centred leadership
2. Person‐centred management practices that improve outcomes	2.1. Patient outcomes
	2.2. Employee outcomes
	2.3. Organizational outcomes
3. Challenges and coping methods for achieving person‐centred management	3.1. Challenges encountered
	3.2. Coping with challenges

#### 4.2.1 Domain 1: Leadership Styles for Person‐Centred Management

This main domain highlights that leadership styles play an integrative role in implementing PCM. Participants' experiences suggest that leadership extends beyond routine administrative tasks, operating instead as a process with empathetic and ethical dimensions. Ultimately, these leadership styles were described as key facilitators for supporting humanistic values within the manager‐employee‐patient triad, perceived as transforming technical care into a person‐centred climate.

##### 4.2.1.1 Subdomain 1.1.: Empathetic Leadership

Participants highlighted that PCM was frequently associated with genuine empathy and compassion. Particularly in chaotic work environments, a leader's capacity to ‘put themselves in the employees' shoes’ was described as important for maintaining the human dimension of management. Ultimately, during times of crisis, this patient and empathetic approach was perceived to extend beyond formal professional boundaries, acting as a stabilizing element.

When I say person‐centred, I am talking about treating others the way we'd like to be treated not just in business, but in every part of life. If we want to see more person‐centred behaviour, we need to model it ourselves. We need to be kind, polite, and courteous…. We should treat others the way we'd like to be treated (P13).

##### 4.2.1.2 Subdomain 1.2.: Ethical Leadership

Participants emphasized that the sustainability of PCM was perceived to depend on ethical leadership characterized by justice, transparency, and merit, rather than strict compliance with rules. Findings suggested that objective evaluations alongside managerial accountability were viewed as essential ethical practices. Furthermore, fostering a blame‐free culture of trust in which errors can be safely disclosed was reported to help employees feel secure and valued, even amid uncertainty

We need to make our employees feel that we're fair. Whether it's an instant reward, fulfilling a special request on their work list, or even just a personal situation, our employees need to know we're fair. They need to be able to come to us with questions and get answers, and we need to be able to justify our actions (P2).

##### 4.2.1.3 Subdomain 1.3.: Person‐Centred Leadership

Participants conceptualized person‐centred leadership as closely linked to the leader's emotional regulation and self‐compassion. To help address burnout and compassion fatigue, the findings underscore the need for concrete organizational strategies (e.g., wellness centres, mindfulness practices), supported by psychological safety and deep listening. Ultimately, this inclusive approach was viewed as a foundational element for supporting a healthy work environment that is perceived to benefit the well‐being of patients, employees, and leaders alike.

A leader who wants to provide person‐centred management should be someone who can recognize and manage their own emotions, value people, strive to listen on a deeper level when listening to others truly, try to understand team members without resorting to violence, ensure psychological safety, show compassion towards others, and most importantly, be compassionate towards themselves (P4).

#### 4.2.2 Domain 2: Person‐Centred Management Practices That Improve Outcomes

The research findings suggest that PCM is not only an administrative but rather a systemic framework perceived to impact all healthcare stakeholders. This overarching domain encapsulates the multifaceted perceived outcomes of PCM, encompassing clinical care experiences and organizational sustainability. Participants reported that this approach was associated with perceived improvements in recovery and patient motivation, was perceived to contribute to a sense of ‘belonging’ and psychological safety among employees and supported a culture of shared responsibility at the organizational level.

##### 4.2.2.1 Subdomain 2.1.: Impact on Patient Outcomes

Participants highlighted that PCM extends beyond technical nursing interventions to broadly address patients' spiritual, cultural, and psychosocial needs. Findings indicated that accommodating religious and cultural values, such as providing sacred texts, was reported to help build trust and satisfaction among patients and their families. Furthermore, integrating care partner programmes and ‘teach‐back’ methods in discharge education was perceived by participants to enhance quality of life and potentially reduce readmissions. Ultimately, this approach was described as shifting patients from passive recipients of care into active clinical partners.

For example, hanging a small picture or symbol appropriate to their religious beliefs in our patients' rooms, or keeping a religious book by their side, greatly motivates them and makes them very happy. We set up a CD player in the room for one of our patients who wanted to listen to the Quran, and this increased both their confidence and satisfaction. The patients' relatives were very happy. Such things have a huge impact on patients and families; you cannot even imagine how grateful they are. For us, this truly exceeds the satisfaction we get from our profession. It affects patients' recovery. It also increases nurses' satisfaction (P8).

##### 4.2.2.2 Subdomain 2.2.: Impact on Employee Outcomes

Participants identified psychological security and a sense of belonging among the outcomes of PCM, with some describing the management culture as a “home” that provides “inner peace.” Accounts indicated that an inclusive climate in which employees feel secure expressing their religious, gender, or personal identities was described as closely linked to enhanced job satisfaction. Additionally, tangible structural supports, such as flexible work arrangements and roster synchronization for academic pursuits, were viewed as practical indicators of the organization's investment in employee development.

…to ensure employee safety and patient safety, we need to consider the needs of every employee. Sometimes it's a simple, preventable mistake, but it can affect the employee psychologically. For this reason, sensitive management and a sensitive hospital culture are very important. I know these things are valued in this hospital, and I like the atmosphere here. Right now, there is a peace here that I cannot describe; it feels like my home. I really like communication among the employees; I have felt this way since my first day at work. Belonging, feeling that sense of belonging, is very important to me. I am happy because I feel it, and I can do my job with peace of mind (P15).

##### 4.2.2.3 Subdomain 2.3.: Impact on Organizational Outcomes

The findings suggest that person‐centeredness may contribute to a culture of quality and accountability. Participants noted that empowering frontline staff to engage with strategic goals (e.g., OKRs) and participate in clinical quality initiatives was perceived to support operational efficiency and organizational commitment. Additionally, this approach was described as extending to a ‘global health’ perspective, where sustainable practices such as waste reduction and rational resource management were viewed as expressions of an ethical responsibility to society and the environment.

At the beginning of each year, the Quality Department asks us about our goals, or we establish OKRs (Objectives and Key Results) with management. At the beginning of the year, I always tell my colleagues, ‘We can all create a departmental goal together’. This way, we can make these goals our own OKRs. When the Quality Department meets, we're always happy to say that all our department's goals are ready! Employees set their own improvement goals. Or, if you prefer, you can track what's happening in the department for a month. Let's consider where we went wrong. So, what can we do about it? After the observation, we work together on an improvement project (P1).

#### 4.2.3 Domain 3: Challenges and Coping Methods for Achieving Person‐Centred Management

Participants described the transition towards PCM as an ongoing process of navigating structural barriers through adaptive coping mechanisms. This domain captures these challenges and the strategies participants used to manage them, which frequently relied on symbolic capital and institutional recognition. The findings suggest that while PCM was described as requiring commitment across all organizational levels, participants viewed interpersonal appreciation and professional validation as essential for its sustainability.

##### 4.2.3.1 Subdomain 3.1.: Challenges Encountered

Participants identified organizational resistance to change and a lack of shared understanding across disciplines as primary barriers to PCM integration, often framing person‐centredness simply as a core professional value. Concerns about base salaries and inconsistent bonus systems were frequently cited as factors perceived to affect nurses' motivation negatively. This challenge was perceived to be compounded by generational differences within the workforce; participants noted that newly recruited nurses often presented with different professional expectations and knowledge bases, highlighting a perceived need for targeted educational support to maintain established PCM standards.

Everyone, from top‐level executives to lower‐level management, doctors, and nurses, must be aware of this process. Let me be clear: this won't work if you pretend to do it. I know that the entire team must be at a certain level. We encounter people who are reluctant and resistant to change. These people are clearly slowing down the process and showing reluctance (P7).

##### 4.2.3.2 Subdomain 3.2.: Coping With Challenges

To address these challenges, participants described organizational strategies rooted in symbolic recognition. They noted that acknowledging personal and professional milestones served as a meaningful source of emotional support. Furthermore, flexibility regarding personal time (e.g., birthday leave) and formal intranet appreciation mechanisms were viewed as practical applications of a person‐centred approach. Accounts suggested that by helping employees feel valued as individuals, these practices were perceived as helping to alleviate burnout and encourage long‐term institutional loyalty.

When I started working here, the most effective time was my birthday. It meant a lot to me that the company recognised it, shared it on the intranet, and that my coworkers sent me emails to celebrate my birthday that day. I have the right to take the day off on my birthday, which is an accepted birthday gift; I am off work that day. I can spend this day with my family however I want… This means that people within the organization see each other not only on birthdays but also on work anniversaries. We receive feedback in our inbox like, “You've been working here for 17 years, congratulations.” I think this is also very valuable for a person‐centred approach (P5).

## 5 Discussion

This study offers an in‐depth exploration of how frontline nurse managers navigate the complexities of PCM in daily clinical practice. By examining leadership styles, stakeholder outcomes, and the structural challenges inherent in implementation, the findings provide a nuanced understanding of PCM beyond its theoretical definition. The results suggest that PCM is not merely a top‐down administrative model but a dynamic process that requires balancing institutional requirements with the humanistic values essential to nursing care.

### 5.1 Leadership Styles for Person‐Centred Management

The leadership styles exhibited by nurse managers are integral to the implementation of PCM. The findings of this study indicate that managers adopt an ethical stance grounded in fairness, objective evaluation, and non‐judgmental communication to foster psychological safety. This aligns with Ayan and Baykal (2024), who underscore the integration of ethical codes. However, the present study adds a practical dimension to the existing body of knowledge by examining how participants described ethical leadership not as a distant professional ideal but as a commitment to equitable treatment regardless of personal or demographic differences. While extant studies frequently associate ethical leadership with broader organizational justice and performance metrics (Sherman et al. 2024; She et al. 2025), our analysis highlights its everyday function in creating a predictable, unbiased environment where staff feel securely represented and heard without fear of judgement.

The participants' accounts also highlight the practical utility of empathy in clinical leadership. While Wibowo and Paramita (2022) theorize a link between empathetic leadership and psychological resilience, our data provide a practical illustration of this relationship through interpersonal interactions. This pragmatic approach to empathy aligns with Östergård et al. (2023), who contend that the nurse‐manager relationship serves as a foundational element in fostering compassionate care. This approach effectively transitions empathy from a passive emotional state to an active problem‐solving instrument.

Our observations regarding people‐centred leadership reveal a strong emphasis on self‐compassion and holistic well‐being as prerequisites for effective management. Although Cardiff et al. (2018) often frame staff involvement as a structural necessity, our participants experienced person‐centred leadership as a process that should begin with the manager's own emotional regulation and self‐actualization. The accounts indicate that proactively addressing compassion fatigue and burnout through both physical and psychological wellness initiatives is perceived not merely as an employee benefit but as a foundational leadership strategy. This focus on sustaining well‐being amidst clinical pressures resonates with Leclerc et al.'s (2021) concept of balancing deeply held leadership values with the structural realities of healthcare systems. It suggests that transitioning to PCM is a continuous practice of aligning personal wellness with professional demands, reflecting the idea that managers may primarily maintain their own well‐being to motivate and support their teams effectively.

### 5.2 Person‐Centred Management Practices That Improve Outcomes

This lived experience of psychological safety aligns with broader evidence demonstrating that inclusive environments are critical in mitigating burnout (Cho and Steege 2025) and act as vital buffers in demanding clinical settings (McCormack and McCance 2016; McCance and McCormack 2025). Ultimately, our findings suggest that when frontline managers actively protect these diverse personal identities, they transform person‐centred management from a mere organizational policy into a tangible safety net that sustains the nursing workforce.

The impact of PCM extends directly to patient care. Existing studies often associate person‐centred interventions with measurable outcomes like safety and patient satisfaction (Rossiter et al. 2020). However, our qualitative findings go a step further by illuminating the complex relational dynamics behind these care processes. This finding suggests that patients who receive treatment using a culturally responsive approach may experience a more supportive healing process. Additionally, the findings of this study demonstrate a reciprocal effect, indicating that observing the positive impact of these personalized interventions on patients and their families also contributes to the nurses' own sense of professional fulfilment.

At the organizational level, the findings suggest that person‐centred practices influence broader institutional strategies and ethical frameworks. A notable aspect of the participants' approach pertains to the conceptualization of environmental sustainability, which encompasses initiatives such as waste reduction and rational resource utilization. These practices are not merely functional; rather, they are ethically grounded, aligning with the principles of PCM and emphasizing a respect for both humanity and nature. This perspective has the potential to broaden the traditional view of organizational outcomes by suggesting that PCM encompasses ecological and ethical responsibilities alongside resource optimization (Pirhonen et al. 2020). Considering the operational challenges associated with high staff turnover in unsupportive work climates, the institutionalization of inclusive strategies is imperative. By cultivating an environment that fosters a sense of integration among staff members in system‐level improvements, organizations can promote professional engagement and develop a more sustainable healthcare delivery model.

### 5.3 Challenges and Coping Methods for Achieving Person‐Centred Management

According to frontline nurse managers, one of the most significant obstacles to PCM is the presence of traditional hierarchical structures. This finding aligns with Leclerc et al.'s (2021) theory that traditional leadership models have reached an impasse. As demonstrated in the extant literature (Howle et al. 2025; Lambert et al. 2024), toxic organizational climates, excessive workloads, and a passive organizational silence environment have been identified as significant obstacles. A salient dimension of these structural impediments pertains to interprofessional dynamics. Frontline nurse managers encounter challenges in addressing interprofessional incompatibilities stemming from physicians' diverse perspectives on person‐centred care (Liang et al. 2023) and in grappling with the inadequacy of staffing policies implemented by senior management. The absence of teamwork and inadequate staffing support, which are essential for nurses to maintain this humanistic model in the field (Baek et al. 2023), represents a pernicious structural impediment to the requirements of PCM.

Despite these challenges, the findings highlight pragmatic coping mechanisms that managers use to cultivate a person‐centred climate. Rather than relying on abstract policies, concepts like structural empowerment are enacted through personalized gestures. By making staff feel visible and appreciated, these managerial touchpoints reflect the healthy work environment models outlined by Choi et al. (2021) and Wang and Dewing (2021), suggesting that trust is built through consistent, humanistic interactions.

By regularly evaluating individual needs and organizing targeted training, managers ensure that innate soft skills are reinforced by continuous professional development. This aligns with Abdelaliem et al. (2025), who argue that personal empathy alone is inadequate without structured educational interventions. Ultimately, the findings suggest that integrating compassion‐based managerial interventions (Nielsen et al. 2025) with transparent, individualized support systems emerges as the viable pathway for institutionalizing PCM amidst systemic barriers.

### 5.4 Strengths and Limitations of the Work

The present study is subject to certain limitations inherent to qualitative inquiry. First, given that the study reflects the experiences of frontline nurse managers in a single private hospital that is highly specialized, ‘Planetree‐certified’ and ‘Joint Commission‐accredited’, the findings are not generalizable but rather transferable to similar contexts. Because this institutional environment inherently prioritizes a person‐centred ethos, participant narratives may be subject to ‘organizational culture bias’ and ‘institutional social desirability’. This context likely predisposes participants to a positivity bias regarding PCM. Consequently, the transferability of these findings to public hospitals, rural facilities, less‐resourced, non‐accredited, or structurally constrained settings may be limited, as the foundational resources that enable these PCM practices may be absent.

A secondary constraint is the reliance on self‐reported data from in‐depth interviews, which introduces potential bias. While this homogeneity is somewhat reflective of the historically female‐dominated culture of the nursing profession, its implications require critical consideration. The prominent emergence of subdomains such as ‘empathetic leadership’ and the high degree of ‘emotional labour’ invested in symbolic appreciation practices may be deeply intertwined with gendered leadership expectations. Female nurse managers are often socially and culturally expected to enact nurturing, emotionally available leadership styles. Consequently, what is observed as PCM might concurrently reflect the disproportionate burden of emotional labour placed upon female leaders within healthcare hierarchies. The absence of male nurse managers in this study precludes an understanding of how gender dynamics might alter the negotiation of power relations, organizational politics, and the enactment of PCM.

Furthermore, conducting ‘insider research’ within the researchers' own institution inherently carries a structural risk of social desirability bias, in which participants might lean towards providing positive accounts. While this remains a limitation of single‐site studies, it was actively mitigated in this research by ensuring that no participants had a direct reporting relationship with the researchers, and by strictly enforcing and frequently reiterating written confidentiality protocols to foster candid discussions.

Notwithstanding the limitations, the study's merits lie in the transparency and systematic nature of its methodology. In the analysis and reporting of the data, a rigorous scientific process was followed to ensure credibility, dependability, and transferability in qualitative research (Polit and Beck 2018; Lincoln and Guba 1986). In this context, the study offers a distinctive contribution to existing literature by presenting the experiences of frontline nurse managers regarding the structural support and person‐centred management necessary for a healthy work environment.

### 5.5 Recommendations for Further Research

Subsequent research endeavours should utilize the qualitative insights derived from this study as a foundational element. Specifically, mixed‐method designs should be employed to assess the relationship between person‐centred management styles and organizational outcomes. While the present study identified perceived benefits for nurses and patients, further research is needed to assess the scope of these impacts fully. To this end, quantitative studies are recommended to assess the statistical correlation between person‐centred leadership behaviours and objective indicators such as nurse turnover rates, patient safety incidents, and quality‐of‐care metrics.

In addition, subsequent research endeavours should prioritize interventional or action research designs to address the specific structural barriers identified in this study. Rather than relying solely on individual‐level training programmes, researchers could develop interventions focused on streamlining bureaucratic workflows and formally integrating relational support systems to transform the identified facilitators into standard institutional policies. A significant contribution to the field would be investigating how these systemic interventions mitigate professional distress, alleviate burnout, and enhance nurse well‐being.

### 5.6 Implications for Policy and Practice

The findings suggest that transitioning from a task‐oriented supervisory model to a relationship‐based leadership approach serves as a highly effective strategy for frontline nurse managers to maintain a desired work environment. Based on the specific leadership and management practices identified in our data; to operationalize these findings, nursing leadership should expand traditional performance metrics to recognize and incentivize relational behaviours explicitly. Since the study findings concluded the importance of diverse leadership styles to facilitate person‐centred management practices, which improves both employee and patient outcomes in the aspects of frontline nurse managers, policy makers and hospital managers may consider adding leadership training programs to their systems for top and frontline managers in order to disseminate the adoption and development of those diverse leadership styles in a unique health care environment.

Considering that the other important conclusion of our study was related to the challenges in implementing person‐centred management and shared the adaptive coping strategies for maintaining person‐centred care in the aspect of frontline nurse managers, explicitly valuing relationship‐building efforts and establishing a supportive culture may be essential for addressing persistent challenges such as nurse burnout, professional distress, and workforce retention.

Furthermore, our findings regarding institutional challenges demonstrate that the sustainable adoption of person‐centred management requires structural support, rather than relying solely on the individual resilience and adaptive coping strategies of frontline managers. As the participants indicated, when nurse managers are overwhelmed by administrative and operational tasks, their capacity to engage in meaningful relationship‐building naturally diminishes. Consequently, nursing administrators should actively streamline bureaucratic workflows, providing frontline managers with the requisite time and operational autonomy to focus on their teams' well‐being. Integrating person‐centred principles into the core operational structure is foundational for supporting both the career longevity of nurse managers and the consistent delivery of person‐centred care.

## 6 Conclusions

This study offers compelling qualitative evidence that the leadership practice of frontline nurse managers is fundamentally anchored in ethical, empathetic, and person‐centred styles. The findings show that when these leadership approaches are implemented, they are perceived to positively influence the entire healthcare process, with participants linking them to supported nurse well‐being, patient safety, and overall organizational quality. By prioritizing staff connection and ethical decision‐making, frontline nurse managers bridge the gap between administrative demands and the realities of bedside care.

In conclusion, cultivating a conducive work environment requires more than individual managerial effort; it demands a systemic commitment to eliminating barriers to person‐centred, ethical and empathetic leadership. Healthcare organizations would benefit from acknowledging that supporting frontline nurse managers in these leadership domains is a strategic priority. Rather than relying solely on individual coping mechanisms, empowering these leaders to overcome structural obstacles is essential to mitigating professional distress and cultivating a sustainable nursing workforce, thereby establishing a foundation perceived to support superior patient and institutional outcomes.

## Author Contributions

**Arzu Kader Harmanci Seren:** conceptualization, writing – review and editing, supervision. **Furkan Cihat Arici:** conceptualization, methodology, data curation, writing – original draft, software.

## Funding

The authors have nothing to report.

## Disclosure

We confirm that AI tools were not used to create, modify, or manipulate any original research data, results, or visualizations, and that no AI technology is listed as an author. The authors maintained full human oversight throughout the preparation of this manuscript and assume full responsibility for the integrity and accuracy of the final submitted work. An earlier version of this study was presented as an e‐poster at the International Council of Nurses (ICN) Congress held in Helsinki, Finland, on June 9–13, 2025.

## Ethics Statement

Ethical approval was obtained from Koç University Ethics Committee (Approval No: 2024.030.IRB3.013, Date: January 26, 2024).

## Conflicts of Interest

The authors declare no conflicts of interest.

## Supporting information


**Supplementary File 1:** COREQ checklist.

**Supplementary File 2:** Additional illustrative quotations based on participants' experiences.

## Data Availability

The data that support the findings of this study are available from the corresponding author.
