# Supplementary material for: Frontline Nurse Managers' Views of Person‐Centred Management in a Private Hospital: A Qualitative Study
Source: Nurs Open. 2026 Aug 13;13(8):e70753. doi: 10.1002/nop2.70753 (PMC13472513; doi:10.1002/nop2.70753)
Supplement: Supplementary file 1 [file NOP2-13-e70753-s001.docx]

Supplementary File 2: Additional Illustrative Quotations Based on Participants' Experiences

| **Domain** | **Subdomain** | **Statements based on participants' experiences.** |
| --- | --- | --- |
| **1. Leadership styles for person-centred management** | **1.1. Empathetic leadership** | *"…. I have to emphasise. When I say "empathy," I'm not just talking about the word, but the kind of empathy that comes from the heart. We really need to practice this. Sometimes we get thrown into chaotic situations, and sometimes we get overwhelmed. I'll admit that. But if you ask me what qualities are necessary for people-oriented management as a manager, I'd say we have to emphasise from the heart and be patient..."* (P10). |
|  |  | *“I always put myself in my team colleagues'. I always think, "How was I when I first started working?" How did I want to be treated? I try to treat my team and colleagues the way I'd want to be treated. To build empathy, I put myself in my nurses' shoes. These days, we have higher expectations of nurses, so I think about what I'd expect from my manager if I were starting out now, and how I'd want them to treat me. I get feedback from them, ask what they want, and try to implement it. I solve problems this way."* (P18). |
|  | **1.2. Ethical leadership** | *"Everything we experience, whether it's right or wrong, needs to be energised by our direct reports to explain it to our managers accurately. Because even the smallest mistake we make, or the right thing to do, can affect everyone. Therefore, employees should be able to speak directly to their managers and know that, if they do, they will be listened to without being judged. I believe managers need to demonstrate to their employees that they have ethical values."* (P21). |
|  |  | *“First, they've got to be objective, fair, and truly just in their work. A leader must be able to connect with everyone on the team. I'm not saying I love the team or that I don't. To make sure management is person-centred, employee differences shouldn't overshadow ethical values. A leader should be able to evaluate employees based on their work. If they keep getting better at their work, they should be given promotions and appointments, no matter what their personality is like, what religion they have, what language they speak, what race they are, or if they're gay or straight.”* (P7). |
|  | **1.3. Person-centred leadership** | *"I believe that a person-centred manager should first value themselves, value the people they work with, the environment they work in, and the surroundings. In other words, they should demonstrate people-oriented leadership qualities and be mindful of themselves, people, animals, and the environment. To ensure people-oriented management, the leader must first feel good about themselves and achieve self-fulfilment so that they can make their team happy with that happiness, provide motivation, and be more effective in terms of contributing."* (P11). |
|  |  | *"We must create this healthy work environment for ourselves, our staff, and our patients; we must find what works for us to be well. For the first time in our country, a wellness centre for both employees and patients have been established at our hospital. XXX is truly an amazing place. I do Pilates and breathing exercises there. I encourage not only myself but also my team to go there frequently. I refer my patients there. For example, we have prepared a program to reduce burnout and compassion fatigue among oncology, haematology, and intensive care nurses. Every Thursday, nurses who are available participate in breathing exercises, receive training on how to approach difficult patients at this centre, do Pilates, participate in body awareness workshops, or do yoga."* (P6). |
| **2. Person-centred management practices that improve outcomes** | **2.1. Patient outcomes** | *“We used to provide nursing care. Patients and their families would come, and we would only provide support during the illness. Now, nurses also focus on improving the patient's quality of life and supporting the post-discharge process. Through our care partner programs, we provide psychological support and involve patients in their care. We also include the patient's family in the process if the patient wishes. Our patient readmission rates are decreasing, and the process at home is easier for them. In discharge education, we use a "teach back" method. Our patients tell us they are satisfied and trust us, and we don't just hear this from our patients; our quality indicators and data prove it.”* (P17). |
|  |  | *“When we created a person-centred climate, we saw that not only did patient satisfaction and quality of care increase, but hospital stays were shortened, and readmission rates decreased. We didn't feel this immediately, but as we continued to foster this climate, we began to see the results.”* (P12). |
|  | **2.2. Employee outcomes** | *“As employees, we feel safer and more valued. I feel comfortable and secure. For example, as a non-Muslim employee, I don't have to hide my religion, nor does another colleague have to hide their sexual identity. We don't have to hide our own characteristics, our own personalities”.* (P8). |
|  |  | *“Sometimes we offer different working models depending on the employee's life situation, for example, part-time work. One of our colleagues takes care of their child with their spouse, who is also a healthcare worker, and working full-time does not allow them to care for their child. So, we offered a part-time work model, management supported this, and we switched to a different work arrangement. This also made things easier for future employees. For instance, there's something this organisation values that I wanted to mention—the schedules of our colleague’s pursuing master's or doctoral degrees are adjusted to align with their school days.”* (P16). |
|  | **2.3. Organizational outcomes** | *“In person-centred management, we consider global health through separation, recycling, and waste reduction. You might think of this as a practice designed to reduce company costs, but it's also about respecting the environment, animals, nature, and people. We've organised training on rational resource use, recruited volunteers for resource management, and implemented numerous projects, such as reducing medical waste.”* (P20). |
|  |  | *“We've been getting our employees more involved in quality processes. We've been having meetings with our managers to chat about how we can improve our approach to preventing falls, reducing pressure sores, and providing effective discharge training. But we've learned that when it comes to person-centred care, it's all about including patients in the process. This has shown us a different path, and we've included our employees in our quality processes. We've had a chat with them about fall processes, asked what they need, and we've all worked together to improve our quality processes.”* (P4). |
| **3. Challenges and coping methods for achieving person-centred management** | **3.1. Challenges encountered** | *“We are facing challenges in providing nurses with adequate salaries. The bonus system is in place, but it's not fixed. When the hospital doesn't make a profit, the nurses aren't given their share. When nurses do not receive any bonuses, their motivation decreases”* (P9). |
|  |  | *“A lot of new nurses want to stick with nursing or not switch careers, so they look for ways to do that. They just aren't that interested in the nursing profession. Or the nurses joining us have different levels of knowledge or professional interests. So, we need to give them more training in professional and person-centred care.”* (P14). |
|  |  | *“Nurses are constantly leaving us for public hospital appointments. Right now, we are losing both our experienced staff and the ones we’ve just hired. When the turnover rate is this high, it becomes incredibly hard to hold onto the existing culture.”* (P7). |
|  | **3.2. Coping with challenges** | *“Every New Year's, I get my team small gifts. I give them all New Year's presents. This makes them very happy, and they feel appreciated. For example, if I see someone working unhappily one day, I'll ask if they have a few minutes to talk. I ask them, "Is there a problem? How can we solve it?" I'm happy to help. I try to boost their morale and improve their daily performance with small touches. Or, if they've got good examples of improvements or care they've provided for the hospital or patients, I'll nominate them for an immediate reward.”* (P19). |
|  |  | *“We use performance management for each employee at our hospital. Every manager has received training on this, and we actively use performance management. Every year, we look at how our employees are doing and what they're capable of. We look at each employee on an individual basis and determine what development and training they need at the end of the year. But our processes weren't like this before. Since switching to this system, we've been meeting regularly with our nurses to discuss what we can do and what they're good at.”* (P3). |
